# Effects of total flavonoids of Rhizoma Drynariae on biochemical indicators of bone metabolism: a systematic review and meta-analysis

**DOI:** 10.3389/fphar.2024.1443235

**Published:** 2024-09-10

**Authors:** Wei Li, Zechen Zhang, Yuyi Li, Zhenyu Wu, Chengjie Wang, Zhen Huang, Baisheng Ye, Xin Jiang, Xiaolong Yang, Xiaolin Shi

**Affiliations:** ^1^ The Second School of Clinical Medicine, Zhejiang Chinese Medical University, Hangzhou, China; ^2^ Department of Orthopaedics, The Second Affiliated Hospital of Zhejiang Chinese Medical University, Hangzhou, China

**Keywords:** osteoporosis, total flavonoids of Rhizoma Drynariae, bone metabolism, bone resorption, bone formation

## Abstract

**Background:**

Evidence shows that the total flavonoids of Rhizoma Drynariae (TFRD) can improve bone mineral density (BMD). However, there is no evidence to summarize the improvement of biochemical indicators of bone metabolism (BIBM).

**Methods:**

The PubMed, Web of Science, Cochrane Library, Embase, Chinese National Knowledge Infrastructure (CNKI), Wanfang Database, Chongqing VIP Information Database (VIP) and SinoMed were searched from inception to 6 May 2024. The final included studies performed meta-analyses using RevMan 5.3.

**Results:**

Nine randomized controlled trials (RCTs) were ultimately included. The TFRD group had higher bone gla protein (BGP) and type I procollagen-N-propeptide (PINP) compared to the Other therapies (WMD: 5.11; 95% CI: 3.37, 6.84; *p* < 0.00001; WMD: 13.89; 95% CI: 11.81, 15.97; *p* < 0.00001). The tartrate-resistant acid phosphatase (TRACP) decreased significantly (WMD: −1.34; 95% CI: −1.62, −1.06; *p* < 0.00001). The alkaline phosphatase (ALP) increased significantly (WMD: 7.47; 95% CI: 6.29, 8.66; *p* < 0.00001). There were no significant differences in serum calcium (SC) or serum phosphorus (SP) levels between the TFRD and control groups (WMD: 0.08; 95% CI: −0.04, 0.20; *p* = 0.17; WMD: 0.02; 95% CI: −0.02, 0.05; *p* = 0.36).

**Conclusion:**

TFRD can stimulate bone formation and prevent bone resorption in osteoporosis (OP) patients, but it has no effect on SC and SP.

**Systematic Review Registration:**

https://www.crd.york.ac.uk/PROSPERO/.

## 1 Introduction

Altered bone microstructure is a hallmark of osteoporosis (OP), a chronic bone disease that weakens the skeleton (Gregson et al., 2022). The pathogenesis of OP can be understood as an abnormality of bone metabolism (BM), where bone resorption (BR) is greater than bone formation (BF), leading to a decrease in bone mass and an imbalance in the skeletal microenvironment. Under normal physiological conditions, bone stabilization consists of a variety of cells that are active in the microenvironment to ensure that BF and BR occur and remain relatively stable to maintain bone mass (Yao et al., 2020). Bone is always in a metabolic state, and the stabilization of BM is mainly dependent on the dynamic balance between osteoblast-mediated BF and osteoclast-mediated BR, which is maintained by the normal bone marrow microenvironment. Deterioration of the bone marrow microenvironment due to aging or hormonal disorders leads to an imbalance between osteoblasts and osteoclasts, which is a key factor in the pathogenesis of OP. According to the 2023 Chinese OP epidemiologic survey, the incidence of OP was 20.73% in males and 38.05% in women, while the prevalence of osteoporotic fracture in the elderly was 18.9% (Wang et al., 2023; Meng et al., 2023). Therefore, timely treatment of OP is essential. Biochemical indicators of bone metabolism (BIBM) includes BF markers, BR markers, and calcium and phosphorus metabolism indicators (CPMI) (Nishizawa et al., 2019; Szulc, 2018). BF markers include alkaline phosphatase (ALP), bone gla protein (BGP), type I procollagen-N-propeptide (PINP), and osteoprotegerin (OPG). BR markers mainly include tartrate-resistant acid phosphatase (TRACP), type I collagen carboxy⁃terminal peptide (CTX), pyridinoline, and deoxypyridinoline. CPMI mainly include SC, SP, parathyroid hormone (PTH), and calcitonin (CT). BGP is a non-collagen protein synthesized by osteoblasts and can reflect the activity of osteoblasts (Cancela et al., 2014). PINP is an extracellular breakdown product of pre-collagen fibers synthesized and released by osteoblasts, which is used to assess the speed of bone turnover markers (BTMs) and type I collagen synthesis, and to reflect collagen synthesis and osteoblast activity (Gillett et al., 2021). Bone alkaline phosphatase (BALP) is an extracellular enzyme that can also reflect osteoblast activity and is a specific marker of BF (Ng et al., 2023). TRACP is mainly derived from osteoclasts and positively correlates with the function of BR, and the level of TRACP in the blood is able to reflect the functional activity of osteoclasts (Solberg et al., 2014). C-terminal telopeptide of type I collagen (β-CTX) is an isomer of the C-terminal peptide of collagen type I, and is a good indicator of BR (Bhattoa et al., 2021). SC and SP are important trace elements required by the human body and are the basis for BF (Ciosek et al., 2021). Therefore, improving BIBM to promote osteoblasts or inhibit osteoclasts can effectively delay the development of OP.

Currently, OP is mainly treated by drugs in clinical practice, including BR inhibitors, BF promoters, and other drugs. Anti-BR drugs mainly inhibit osteoclast BR, including bisphosphonates, calcitonin, estrogen, and so on. Bisphosphonates can increase the level of BM and effectively reduce the risk of osteoporotic fracture (Rhee et al., 2006; Nakamura et al., 2021), but prolonged use of bisphosphonates increases the risk of osteonecrosis of the jaw and atypical femur fracture (Zhang et al., 2018; Black et al., 2020), and long-term use is not recommended. A hormone that controls calcium levels called calcitonin has the ability to reduce osteoclasts’ biological activity and bone loss, and alleviate bone pain, but there is a possibility that nasal spray salmon calcitonin may increase the risk of tumors, and the duration of use is limited (Kaskani et al., 2005). Estrogen is effective in reducing bone loss and decreasing the risk of fracture in postmenopausal women, but increases the risk of endometrial cancer and breast cancer (Wu et al., 2018; Vinogradova et al., 2020). BF promoters mainly stimulate osteoblastic BF, including parathyroid hormone analogs, such as teriparatide. Teriparatide stimulates osteoblast activity, promotes BF, and increases bone mineral density (BMD). However, studies have shown that there may be a risk of osteosarcoma, so the duration of treatment was limited (Kendler et al., 2018; Boonen et al., 2008). Other drugs refer to traditional Chinese medicine (TCM) treatments, commonly used TCM include Xianling Gubao capsules, Gushukang capsules and JinTiange capsules, etc. The more clearly defined active ingredients in the medications used are the total flavonoids of Rhizoma Drynariae (TFRD), the total flavonoids of Epimedium, and the artificial tiger bone powder. TCM has the advantages of being safe, inexpensive, and without obvious side effects, so it is getting more and more attention in China. In recent years, more and more studies have demonstrated the effectiveness of TCM in the treatment of OP. Zhu showed that Xianling Gubao capsules could improve BMD without adverse effects through a study (Zhu et al., 2012). Through a study, Liang proved that Jintiange capsules might successfully lower the risk of falls in patients with OP by improving muscle strength and balance (Liang et al., 2022). Currently, studies are increasingly focusing on the treatment of OP from herbal monomers. Total flavonoids from Epimedium can regulate osteogenic differentiation and adipogenesis in mesenchymal stem cells, thus promoting bone growth (Chen et al., 2022; Zhang et al., 2008; Zhang et al., 2016). The components of Qianggu capsules are TFRD, and studies have demonstrated that TFRD can improve the antioxidant capacity, activate the Wnt/β-catenin signaling pathway, promote osteoblastic activity, reduce bone mineral loss, and increase BMD (Shen et al., 2020; Li et al., 2021; Mu et al., 2021). A meta-analysis was conducted to assess the effectiveness of TFRD in OP; however, no full meta-analysis has been conducted to evaluate its BIBM (Wei et al., 2017). Therefore, the present study evaluated the efficacy of TFRD on the BIBM of OP patients by including clinical randomized controlled trials (RCTs) with the aim of obtaining higher-quality clinical evidence.

## 2 Methods

This meta-analysis adhered to the Preferred Reporting Items for Systematic Reviews and Meta-Analyses (PRISMA) standards (Page et al., 2021) and is registered with PROSPERO (CRD42024545497).

### 2.1 Eligibility criteria

#### 2.1.1 Inclusion criteria

(a): The study included only patients with a diagnosis of OP, including postmenopausal osteoporosis (PMOP) and senile osteoporosis (SOP). Referring to the diagnostic criteria recommended by the WHO, for postmenopausal women and men aged 50 years and above, T score ≤ −2.5 on dual-energy X-ray absorptiometry (DXA) of the BMD of the mid-axis bones (lumbar 1-4, femoral neck, or total hip) or of the distal one-third of the radius is considered to be OP.
(b):The study compared TFRD to other methods of OP treatment.
(c): The study included at least one of the following assessment instruments: BGP, PINP, TRACP, SC, SP, ALP.
(d): The study was RCTs.

#### 2.1.2 Exclusion criteria

(a): Animal studies, reviews, meta-analyses, etc.
(b): Duplicate publication.
(c): Patients with OP and other diseases.
(d): The studies in which raw data were lacking or data couldn’t be extracted.

### 2.2 Literature search strategy

The PubMed, Web of Science, Cochrane Library, Embase, CNKI, Wanfang Database, VIP and SinoMed were searched. The time frame for the search was from database creation to 6 May 2024. The search strategy was conducted using a combination of free words and MeSH terms. The relevant keywords for OP were “osteoporosis” OR “senile osteoporosis” OR “postmenopausal osteoporosis” OR “primary osteoporosis.” The keywords of TFRD, including “Total Flavonoids of Rhizoma Drynariae” OR “Qianggu capsule” OR “Qianggu jiaonang.” Each database’s unique qualities informed the search approach, as detailed in the Supplementary Material.

### 2.3 Data extraction and biased risk assessment

Author names, year of publication, sample size, number of participants in experimental and control groups, mean age of participants, country, intervention and control, duration of treatment, and outcome measures were retrieved by two authors from the nine studies.

Using the Physiotherapy Evidence Database (PEDro) scale, two authors evaluated the risk of bias in the included research (Cashin and McAuley, 2020). A third assessor conducted arbitration or discussions to resolve disagreements. The total PEDro score was obtained by summing the scores for items 2 through 11, which ranged from 0 to 10. Elevated ratings signify superior methodological quality. A score of less than four was regarded as “poor,” four to five as “fair,” six to eight as “good,” and nine to ten as “excellent.”

### 2.4 Outcome measures

The primary outcome measures for this meta-analysis were BF, including BGP and PINP. The secondary measures were BR and CPMI, including TRACP, SC, SP, and ALP.

### 2.5 Data analysis

Data were analyzed using RevMan software (Version 5.3). 95% confidence intervals (CI) of the combined mean difference (MD) were used to assess differences in outcomes between patients with OP who received TFRD and those who received other forms of treatment. The chi-square test was used to analyze and quantify the magnitude of heterogeneity in the included studies. I^2^ > 50%, heterogeneity exists and the source of heterogeneity needs to be analyzed. If I^2^ < 50%, no heterogeneity exists, indicating a good concordance of results. In addition, it is necessary to choose the appropriate method of combining effect sizes according to the magnitude of heterogeneity. The meta-analysis was conducted using a fixed effects model (I^2^ < 50%) if the heterogeneity was small, or a random effects model if the heterogeneity was high (I^2^ > 50%). At *P* < 0.05, statistical significance was taken into account.

## 3 Results

### 3.1 Search result

**FIGURE 1:**
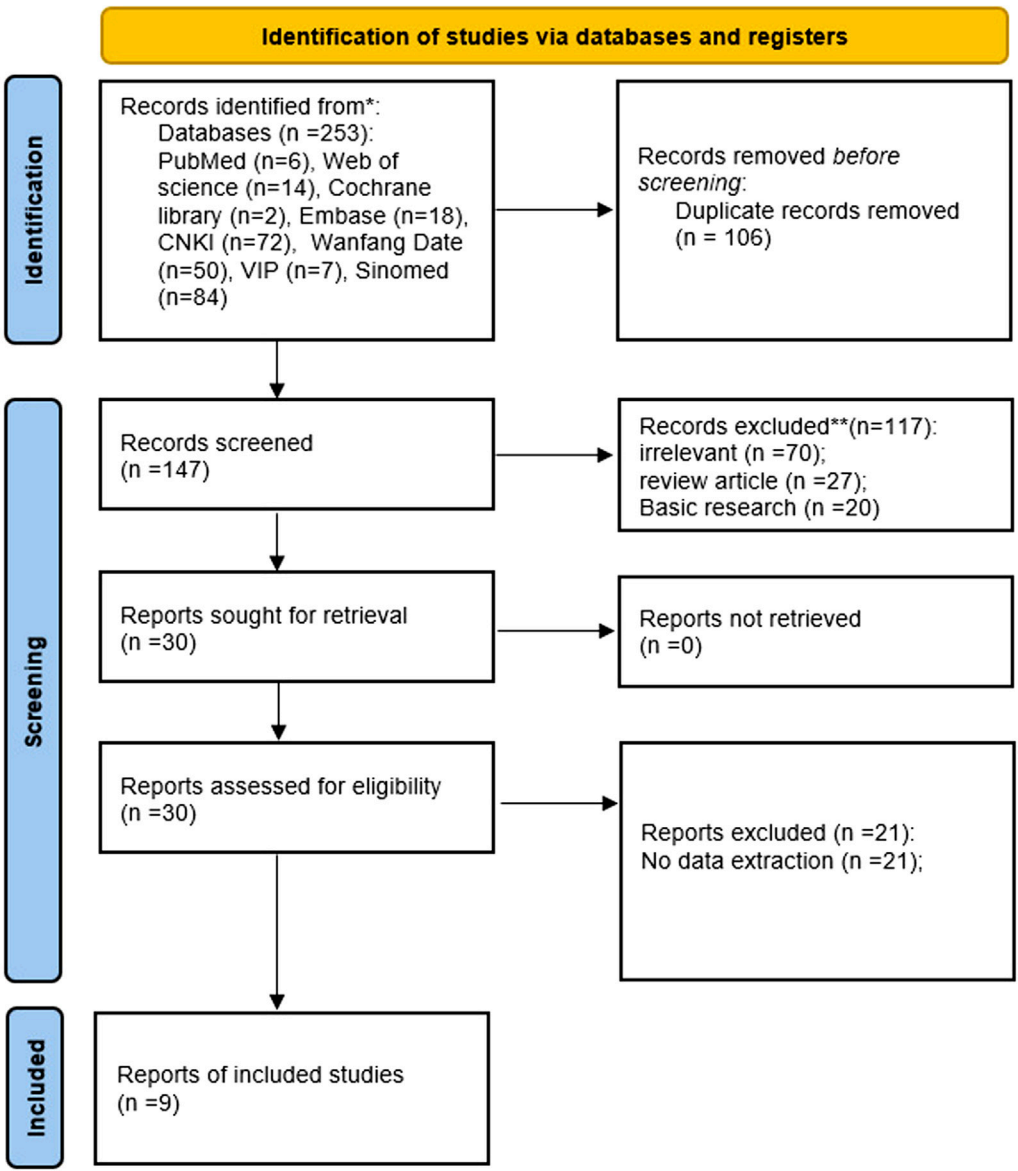
Flowchart of the study selection process.

With eight databases, a total of 253 studies related to the treatment of OP with TFRD were included. Nine studies were ultimately included according to the criteria for inclusion and exclusion (Bian, 2016; He, 2005; Shan and Zhou, 2006; Shen et al., 2018; Wang et al., 2007; Wen and Zhou, 2015; Yang et al., 2017; Zhang and Chen, 2008; Zhou et al., 2017) (Figure 1).

### 3.2 Study characteristics

There were 777 participants in nine investigations, and the sample sizes varied from 46 to 123. Five studies were monotherapy (TFRD), and four were combination therapy (TFRD + C). The treatment duration was a minimum of 4 weeks and a maximum of 6 months. Seven studies used SC, SP, and ALP as outcome measures. Two studies used BGP, PINP as outcome measures. Four studies used TRACP as an outcome measure (Table 1).

**TABLE 1:** The basic characteristics of included studies.

References	Sample size	T/C (M/F)	Age (years)	Country	Intervention	Control	Treatment duration	Outcome measures
Bian (2016)	123	T: 68 (36/32) C: 55 (31/24)	T: 69.8 ± 9.28 C: 69.13 ± 9.02	China	TFRD + C	Calcitriol soft capsules + Calcium carbonate and vitamin D3 tablets	3 months	a, b, c, f
He (2005)	110	T: 52 (18/34) C: 58 (26/32)	—	China	TFRD	Gusongbao granulas	6 months	a, b, c
Shan and Zhou (2006)	62	T: 32 (13/19) C: 30 (12/18)	T: 60.32 ± 4.58 C: 60.96 ± 5.06	China	TFRD	Alfacalcidol soft capsules	3 months	a, b, c
Shen et al. (2018)	100	T: 50 (26/24) C: 50 (23/27)	—	China	TFRD + C	Wei D2 Lin Pu Gai Pian	6 months	c, d, e, f
Wang et al. (2007)	54	T: 28 C: 26	T: 61.8 ± 6.1 C: 62.3 ± 5.9	China	TFRD	Alfacalcidol soft capsules	6 months	a, b, c
Wen and Zhou (2015)	120	T: 60 (33/27) C: 60 (34/26)	T: 69.7 ± 9.3 C: 69.2 ± 9.1	China	TFRD + C	Calcitriol soft capsules + Calcium carbonate and vitamin D3 tablets	3 months	a, b, c, f
Yang et al. (2017)	46	T: 23 C: 23	—	China	TFRD	Alendronate sodium tablets	6 months	a, b
Zhang and Chen (2008)	60	T: 37 C: 23	—	China	TFRD	Guhe injection	3 months	a, b
Zhou et al. (2017)	102	T: 51 (25/26) C: 51 (23/28)	T: 65.43 ± 1.34 C: 65.31 ± 1.22	China	TFRD + C	Cervus and cucumis polypeptide for injection	4 weeks	c, d, e, f

a, SC; b, SP; c, ALP; d, BGP; e, PINP; f, TRACP; C, control; F, female; M, man; T, treatment; TFRD, total flavonoids of Rhizoma Drynariae.

### 3.3 Risk of bias assessment

The quality of the included studies were independently evaluated by WL and YZW using the PEDro tool. The included studies had a mean PEDro score of 6.33. All nine studies had a score greater than 6, indicating a low risk of bias (Table 2).

**TABLE 2:** Physiotherapy Evidence Database (PEDro) scores of the 9 included studies.

References	Eligibility criteria	Random allocation	Concealed allocation	Baseline comparability	Blind subjects	Blind therapists	Blind assessor	Adequate follow-up dropout < 15%	Intention-to- treat analysis	Between- group comparisons	Point estimates and variability	Score
Bian (2016)	1	0	0	1	0	0	0	1	1	1	1	5
He (2005)	1	0	0	1	0	0	0	1	1	1	1	5
Wang et al. (2007)	1	1	1	1	0	0	0	1	1	1	1	7
Wen and Zhou (2015)	1	1	1	1	0	0	0	1	1	1	1	7
Shan and Zhou (2006)	1	1	1	1	0	0	0	1	1	1	1	7
Shen et al. (2018)	1	1	1	1	0	0	0	1	1	1	1	7
Yang et al. (2017)	1	1	1	1	0	0	0	1	1	1	1	7
Zhang and Chen (2008)	1	1	1	1	0	0	0	1	1	1	1	7
Zhou et al. (2017)	1	0	0	1	0	0	0	1	1	1	1	5
Mean												6.33

### 3.4 Meta-analysis results

#### 3.4.1 BF

##### 3.4.1.1 BGP

**FIGURE 2:**
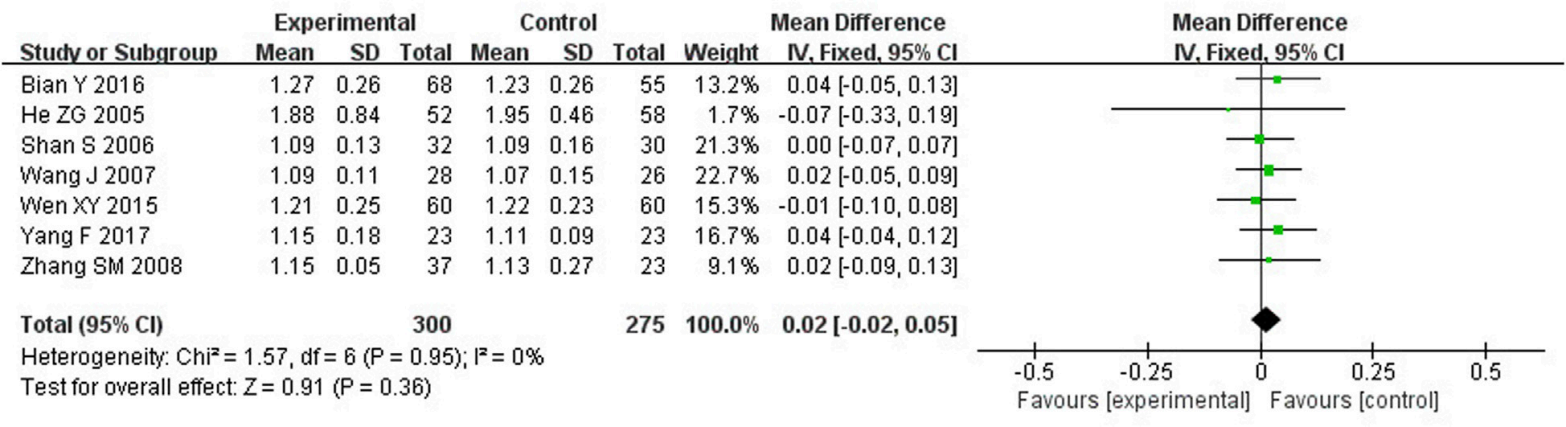
Effect of TFRD on BGP.

BGP were recorded for 202 patients in two studies. The TFRD group had significantly higher BGP levels, according to the meta-analysis (WMD: 5.11; 95% CI: 3.37, 6.84; *p* < 0.00001). The meta-analysis showed that TFRD improves BGP in OP patients more than other therapies (Figure 2).

##### 3.4.1.2 PINP

**FIGURE 3:**
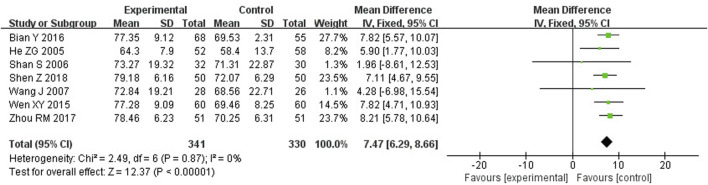
Effect of TFRD on PINP.

The meta-analysis revealed that TFRD was statistically significant in improving PINP in patients with OP. PINP indicators were described in 2 studies, which included 202 patients. The PINP was improved in the TFRD group (WMD: 13.89; 95% CI: 11.81, 15.97; *p* < 0.00001) (Figure 3).

#### 3.4.2 BR

##### 3.4.2.1 TRACP

**FIGURE 4:**
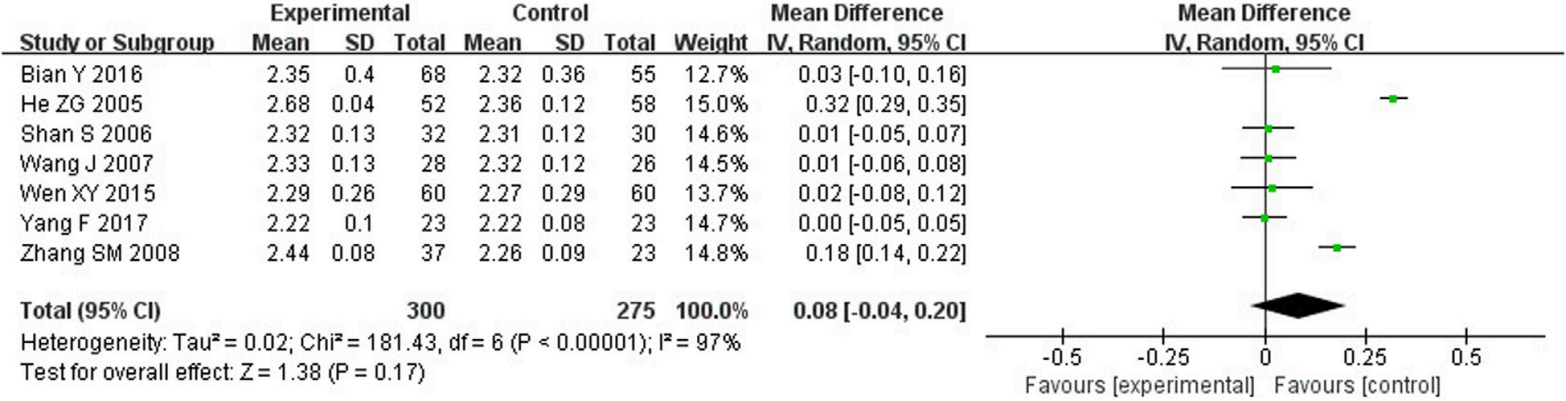
Effect of TFRD on TRACP.

The findings of the meta-analysis indicated that TFRD was statistically significant in improving TRACP in OP patients. TRACP indicators were described in 4 studies, which included 445 patients. TRACP was significantly lower in the TFRD group (WMD: −1.34; 95% CI: −1.62, −1.06; *p* < 0.00001). However, there was high heterogeneity in the results (I^2^ = 91%) (Figure 4).

#### 3.4.3 CPMI

##### 3.4.3.1 SC and SP

**FIGURE 5:**
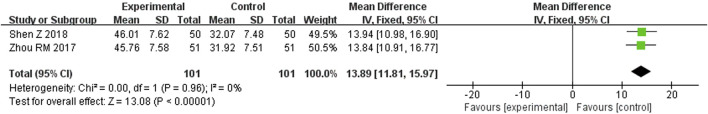
Effect of TFRD on SC.

**FIGURE 6:**
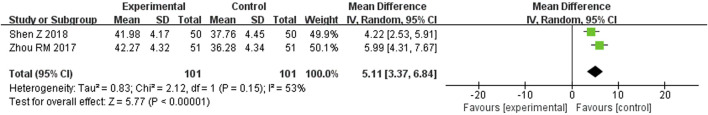
Effect of TFRD on SP.

Meta-analysis showed that TFRD was not statistically significant in improving SC and SP in OP patients. SC and SP were described in 7 studies, including 575 patients. Compared to the other treatment, there was no difference in SC and SP (WMD: 0.08; 95% CI: −0.04, 0.20; *p* = 0.17; WMD: 0.02; 95% CI: −0.02, 0.05; *p* = 0.36) (Figures 5, 6).

##### 3.4.3.2 ALP

**FIGURE 7:**
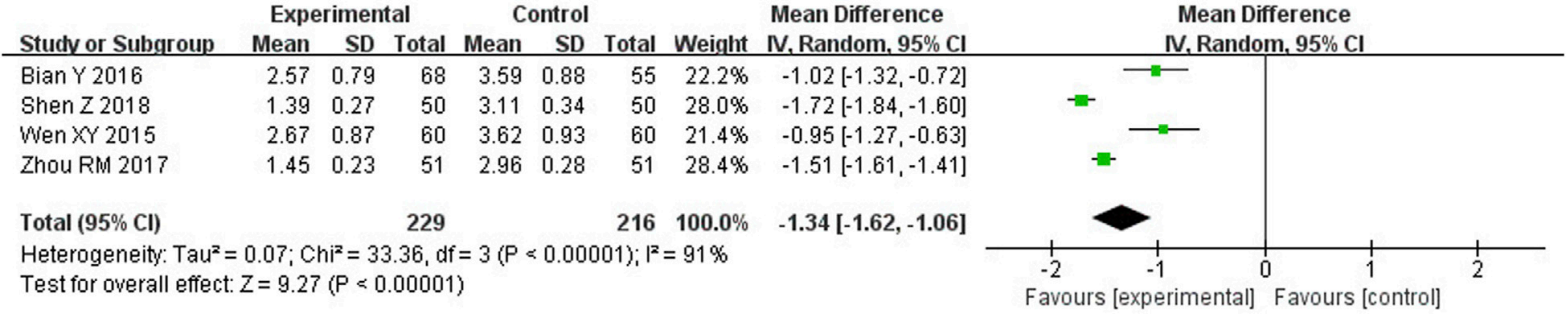
Effect of TFRD on ALP.

Meta-analysis showed that TFRD improves ALP in patients with OP. ALP was described in 7 studies, including 671 patients. ALP was significantly improved in the TFRD group (WMD: 7.47; 95% CI: 6.29, 8.66; *p* < 0.00001) (Figure 7).

#### 3.4.4 Publication bias

**FIGURE 8:**
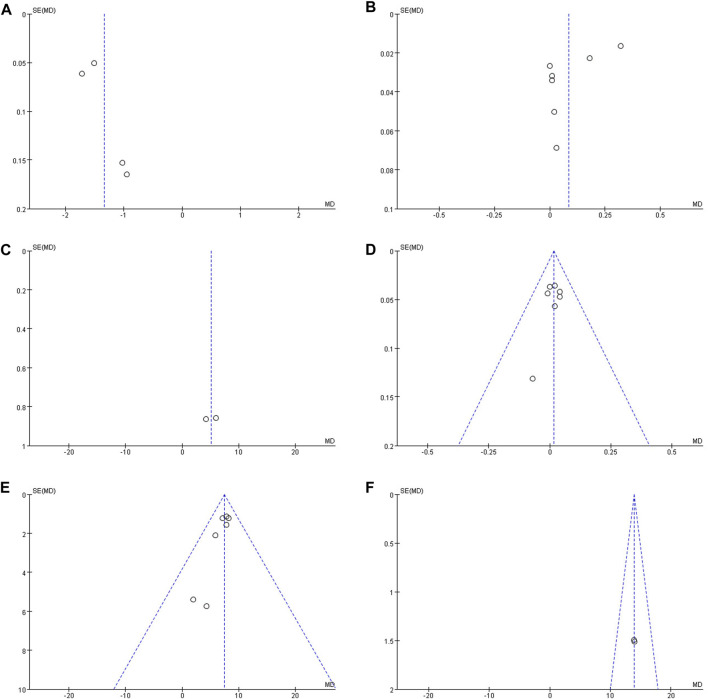
**(A)** Funnel plot of BGP; **(B)** Funnel plot of PINP; **(C)** Funnel plot of TRACP; **(D)** Funnel plot of SC; **(E)** Funnel plot of SP; **(F)** Funnel plot of ALP.

Publication bias was analyzed for all of the outcome measures, and the funnel plots showed that the outcome measures were largely symmetrical and there was no publication bias (Figure 8).

## 4 Discussion

OP contributes significantly to the occurrence of fractures in the elderly. With the aging of the population, the prevalence of OP and osteoporotic fractures is increasing. Postmenopausal women have a higher risk of fracture due to the imbalance in BTMs and severe bone loss as a result of age and hormonal changes in the body (Garnero et al., 2000). Therefore, preventing fractures in OP patients is the current focus. Currently, BMD is commonly used to assess the risk of fracture in patients with OP. Although BMD has been recognized as the gold standard for OP diagnosis, BMD is generally found to change significantly only in 1–2 years, which is not a good assessment of the risk of osteoporotic fracture and is not suitable for short-term follow-up (Deal, 2001; Syed and Khan, 2002). BIBM, as a highly sensitive monitoring index, can well reflect the status of BM at different stages and the efficacy of OP treatment (Biver et al., 2012; Kanis et al., 2000; Khashayar et al., 2015). Several guidelines around the world recommended the use of PINP and β-CTX for OP screening or monitoring treatment (Compston et al., 2017; Cosman et al., 2014; Kanis et al., 2008). There was a study that investigated the association between BTMs and fracture incidence in postmenopausal and older women and found that higher levels of BTMs were associated with a higher risk of fracture (Vasikaran et al., 2011). A meta-analysis demonstrated that PINP and β-CTX appeared to predict fracture risk independently of BMD (Tian et al., 2019). Further studies have shown that BTMs are better at predicting fractures in the short term (Ivaska et al., 2010; Robinson-Cohen et al., 2011). Therefore, BIBM proved valuable in the evaluation of patients with OP.

TCM has obvious advantages in improving patients’ clinical symptoms. With the ability to enhance microcirculation and nourish bones, Rhizoma Drynariae was a popular kidney tonic herb in TCM. Its extract, TFRD, was extensively utilized in the treatment of OP. Modern pharmacological trials have concluded that the TFRD can regulate the levels of cytokines and hormones in the process of BM, inhibit BR, and have anti-inflammatory, analgesic, and fracture healing effects (Jin et al., 2022; Lin et al., 2022; Zhao et al., 2021). A study showed that after 10 weeks of continuous administration of RDTF alone or the combination of RDTF and CaCO_3_ in ovariectomized rats, it was able to improve the trabecular thickness and trabeculae microstructure, and that the combination of RDTF and CaCO_3_ increased the level of ALP and significantly decreased TRACP and S-CTX, which was able to improve the BM, and had a better effect on the repair of fractures (Hu et al., 2021). The combination of estrogen and estrogen receptors can affect the cell cycle, induce osteoclast apoptosis, inhibit osteoclast activity and differentiation, and ultimately inhibit osteoclast BR and prevent OP. Naringin and naringenin were the main active components of TFRD. Studies have shown that naringin and naringenin have estrogen-regulating effects and prevent OP through estrogen receptor agonism (Guo et al., 2011). The Wnt/β-catenin signaling pathway can regulate bone-related bioinformatic signaling, and plays an important role in regulating osteogenic differentiation and BF of bone marrow mesenchymal stem cells (BMSCs). Naringenin can induce the differentiation of BMSCs to osteoblasts under the induction of the Wnt/β-catenin signaling pathway, which was a relevant target for the treatment of OP with TFRD (Yang et al., 2022). In addition, naringin could significantly upregulate the expression of Notch1 in BMSCs and promote osteogenic differentiation of BMSCs by activating the Notch signaling pathway (Yu et al., 2016). TFRD has been demonstrated in clinical trials to be useful in the management of OP. There were only two systematic reviews to evaluate the efficacy of TFRD in the treatment of OP (Wei et al., 2017; Zhang et al., 2017). However, the reviews evaluated TFRD to improve BMD, but there was no BM. Therefore, we analyzed and summarized the level of BIBM improvement by TFRD and performed a meta-analysis.

A total of nine papers were included in this review to analyze the effect of TFRD. The meta-analysis showed that both BF and BR were significantly improved in OP treated with TFRD compared with the control group, suggesting that TFRD was able to increase BGP, PINP, and ALP and to decrease TRACP. However, there was no significant improvement for SC and SP. Although there was high heterogeneity in the results for TRACP and SC, subgroup analysis was not possible because of the short number of included studies. Consequently, we reasoned that the heterogeneity could be a result of the few included studies.

There were limitations of this study. First, only nine randomized controlled trials were included in this study, all of which were single-center clinical studies and lacked multicenter and large sample sizes. Second, the clinical trials jointly used fewer outcome indicators to observe the effect of TFRD on BM from more perspectives. Third, there was a high degree of heterogeneity in some of the outcomes, however, due to the small amount of literature included, subgroup analyses could not be performed, and although we performed publication bias analyses, they do not fully represent the stability of the results, and therefore more studies were needed in the future.

## 5 Conclusion

By including RCTs, our meta-analysis suggested that TFRD may promote BF and inhibit BR, but the number of studies was small, and further evaluation was needed.

## Data Availability

The original contributions presented in the study are included in the article/Supplementary Material, further inquiries can be directed to the corresponding author.
