# Supplementary material for: Effects of total flavonoids of Rhizoma Drynariae on biochemical indicators of bone metabolism: a systematic review and meta-analysis
Source: Front Pharmacol. 2024 Sep 10;15:1443235. doi: 10.3389/fphar.2024.1443235 (PMC11445651; doi:10.3389/fphar.2024.1443235)
Supplement: Supplementary file 1 [file DataSheet1.DOCX]

**Literature search strategy**

**Pubmed**

**(1 OR 2) AND 3**

**1.Search: (((((((((((((((("Osteoporosis"[Mesh]) OR (Osteoporosis, Senile[Title/Abstract])) OR (Osteoporoses, Senile[Title/Abstract])) OR (Senile Osteoporoses[Title/Abstract])) OR (Osteoporosis, Involutional[Title/Abstract])) OR (Senile Osteoporosis[Title/Abstract])) OR (Osteoporosis, Age-Related[Title/Abstract])) OR (Osteoporosis, Age Related[Title/Abstract])) OR (Bone Loss, Age-Related[Title/Abstract])) OR (Age-Related Bone Loss[Title/Abstract])) OR (Age-Related Bone Losses[Title/Abstract])) OR (Bone Loss, Age Related[Title/Abstract])) OR (Bone Losses, Age-Related[Title/Abstract])) OR (Age-Related Osteoporosis[Title/Abstract])) OR (Age Related Osteoporosis[Title/Abstract])) OR (Age-Related Osteoporoses[Title/Abstract])) OR (Osteoporoses, Age-Related[Title/Abstract])**

**2.Search: ((((((((((((((((("Osteoporosis, Postmenopausal"[Mesh]) OR (Perimenopausal Bone Loss[Title/Abstract])) OR (Bone Loss, Postmenopausal[Title/Abstract])) OR (Bone Losses, Postmenopausal[Title/Abstract])) OR (Postmenopausal Bone Losses[Title/Abstract])) OR (Osteoporosis, Post-Menopausal[Title/Abstract])) OR (Osteoporoses, Post-Menopausal[Title/Abstract])) OR (Osteoporosis, Post Menopausal[Title/Abstract])) OR (Post-Menopausal Osteoporoses[Title/Abstract])) OR (Post-Menopausal Osteoporosis[Title/Abstract])) OR (Postmenopausal Osteoporosis[Title/Abstract])) OR (Osteoporoses, Postmenopausal[Title/Abstract])) OR (Postmenopausal Osteoporoses[Title/Abstract])) OR (Bone Loss, Perimenopausal[Title/Abstract])) OR(Bone Losses, Perimenopausal[Title/Abstract])) OR (Perimenopausal Bone Losses[Title/Abstract])) OR (Postmenopausal Bone Loss[Title/Abstract])) OR (primary osteoporosis[Title/Abstract])**

**3.Search: ((****Total Flavonoids of Rhizoma Drynariae[Title/Abstract]) OR (****Qianggu capsule[Title/Abstract])) OR (****Qianggu jiaonang[Title/Abstract])**

**Web of science**

**TS=(Osteoporosis OR Senile Osteoporosis OR Age-Related Osteoporosis OR Age-Related Bone Loss OR Postmenopausal Osteoporosis OR Postmenopausal Bone Loss OR primary osteoporosis) AND TS=(****Total Flavonoids of Rhizoma Drynariae OR Qianggu capsule OR** **Qianggu jiaonang) AND TS=(randomized controlled trial OR controlled clinical trial OR random allocation OR double-blind OR single-blind OR Randomly OR randomized OR clinical trial OR trial OR RCT)**

**Embase**

**#1 AND #2**

**#1. 'umbilical cord mesenchymal stem cells':ab,ti OR 'umbilical cord blood mesenchymal stem cells':ab,ti OR 'umbilical cord-derived stem cells':ab,ti OR 'human umbilical cord mesenchymal stem cells':ab,ti OR 'human umbilical cord blood mesenchymal stem cells':ab,ti OR 'human umbilical cord-derived mesenchymal stem cells':ab,ti OR 'uc mscs':ab,ti OR 'huc mscs':ab,ti**

**#2. 'Total Flavonoids of Rhizoma Drynariae':ab,ti OR 'Qianggu capsule':ab,ti OR 'Qianggu jiaonang':ab,ti**

**Cochrane library**

**#8 AND #12**

**#1: Osteoporosis; #2: Senile Osteoporosis; #3: Age-Related Osteoporosis #4: Age-Related Bone Loss; #5: Postmenopausal Osteoporosis; #6: Postmenopausal Bone Loss; #7: primary osteoporosis (#8: #1 OR #2 OR #3 OR #4 OR #5 OR #6 OR #7)**

**#9: Total Flavonoids of Rhizoma Drynariae; #10: Qianggu capsule; #11: Qianggu jiaonang (#12: #9 OR #10 OR #11)**

**CNKI**

骨碎补总黄酮 + 强骨胶囊；骨质疏松 + 骨质疏松症 + 骨痿；随机对照 + 随机对照试验 + 随机对照研究 + 临床观察 + 临床研究 + 临床疗效

**WanFang**

((题名或关键词:(骨质疏松) or 题名或关键词:(骨质疏松症) or 题名或关键词:(骨痿)) and (题名或关键词:(骨碎补总黄酮) or 题名或关键词:(强骨胶囊))) and (题名或关键词:(随机对照) or 题名或关键词:(随机对照试验) or 题名或关键词:(随机对照研究) or 题名或关键词:(临床观察) or 题名或关键词:(临床研究) or 题名或关键词:(临床疗效))

**SinoMed**

("骨质疏松症"[常用字段:智能] OR "年龄相关骨质疏松"[常用字段:智能] OR "年龄相关骨质丢失"[常用字段:智能] OR "老年性骨质疏松"[常用字段:智能] OR "绝经后骨质丢失"[常用字段:智能] OR "绝经后骨质疏松"[常用字段:智能] OR "绝经期骨质丢失"[常用字段:智能] OR "闭经后骨质疏松"[常用字段:智能]) AND ("随机对照试验"[常用字段:智能] OR "随机对照研究,"[常用字段:智能] OR "临床试验,"[常用字段:智能] OR "临床观察"[常用字段:智能] OR "临床研究"[常用字段:智能] OR "临床疗效"[常用字段:智能]) AND ("骨碎补总黄酮"[常用字段:智能] OR "强骨胶囊"[常用字段:智能])

**VIP**

((K=(骨质疏松症 OR 骨质疏松 OR 骨痿) AND (K=骨碎补总黄酮 OR 强骨胶囊)) AND (K=随机对照试验 OR 随机对照研究 OR 临床观察 OR 临床研究 OR 临床疗效))
